# Supplementary material for: A single-cell transcriptional landscape of immune cells shows disease-specific changes of T cell and macrophage populations in human achalasia
Source: Nat Commun. 2023 Aug 4;14:4685. doi: 10.1038/s41467-023-39750-5 (PMC10403544; doi:10.1038/s41467-023-39750-5)
Supplement: Supplementary file 1 — Supplementary Information [file 41467_2023_39750_MOESM1_ESM.pdf]

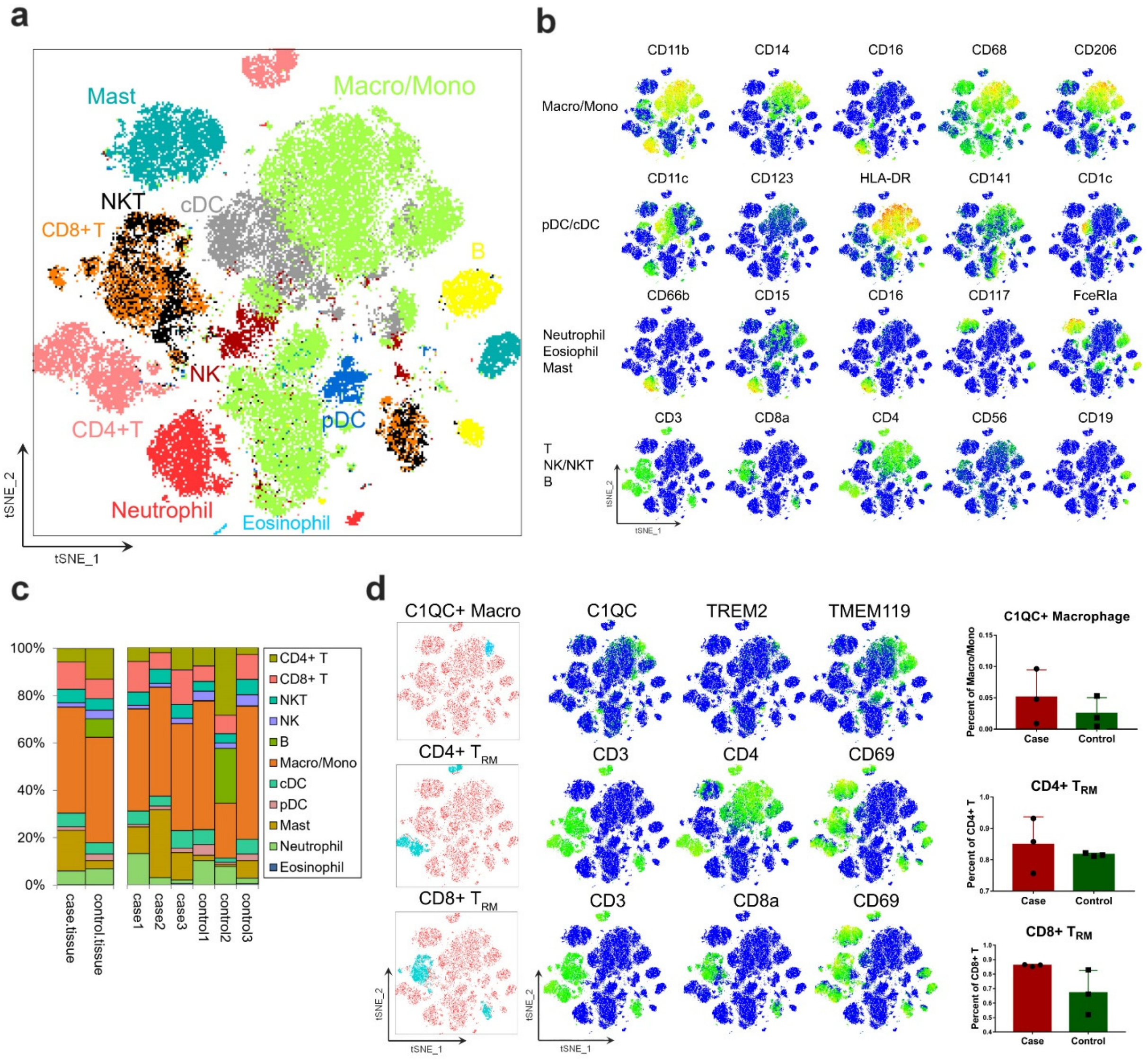

Supplementary Figure 1 cyTOF analysis of immune cells in the LES tissue. a, t-SNE plot of the major immune cells found in the LES tissue by cyTOF analysis. b, t-SNE plots of canonical marker genes for the major immune cells found in the LES tissue. c, Major immune cell proportions for LES tissue and blood between achalasia and controls, colored by cell type. d, t-SNE plots (left), marker genes (middle), and quantifications (right) of C1QC^{+} macrophages and T_{RM} in CyTOF analysis. The quantifications were compared between achalasia (n = 3) and controls (n = 3), and data are represented as mean ± SD. Statistics: two-tailed unpaired t test. CyTOF analysis showed an increased tendency of C1QC^{+} macrophages and T_{RM} in the LES of achalasia

compared with controls. Macro/Mono, macrophages and monocytes. Source data are provided as a Source Data file.

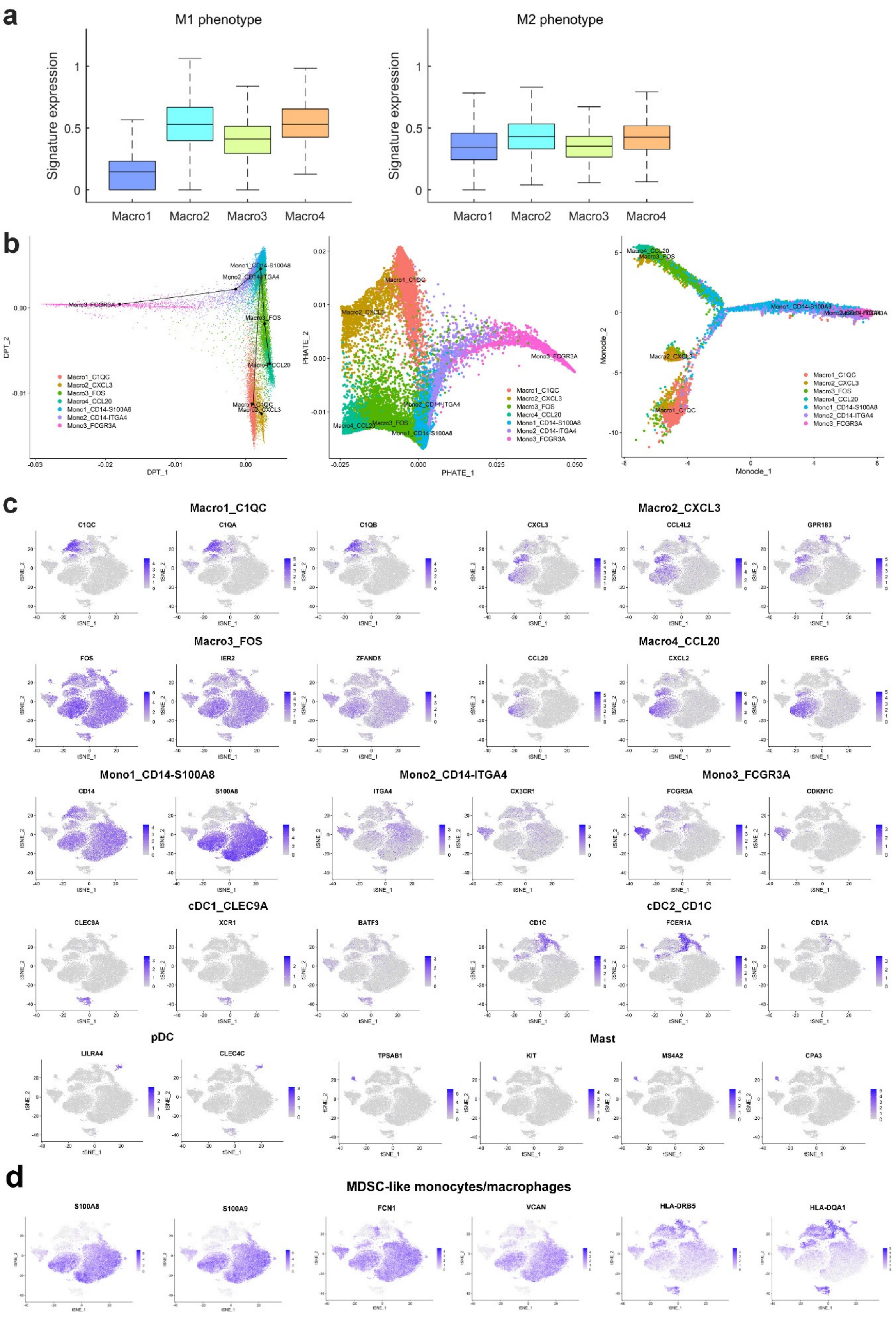
a
M1 phenotype
M2 phenotype
Signature expression
Macro1
Macro2
Macro3
Macro4
b
DPT_1
DPT_2
PHATE_1
PHATE_2
Monocle_1
Monocle_2
Macro1_C1QC
Macro2_CXCL3
Macro3_FOS
Macro4_CCL20
Mono1_CD14-S100A8
Mono2_CD14-ITGA4
Mono3_FCGR3A
c
Macro1_C1QC
C1QC
C1QA
C1QB
Macro2_CXCL3
CXCL3
CCL4L2
GPR183
Macro3_FOS
FOS
IER2
ZFAND5
Macro4_CCL20
CCL20
CXCL2
EREG
Mono1_CD14-S100A8
CD14
S100A8
Mono2_CD14-ITGA4
ITGA4
CX3CR1
Mono3_FCGR3A
FCGR3A
CDKN1C
cDC1_CLEC9A
CLEC9A
XCR1
BATF3
cDC2_CD1C
CD1C
FCER1A
CD1A
pDC
LILRA4
CLEC4C
Mast
TPSAB1
KIT
MS4A2
CPA3
tSNE_1
tSNE_2
d
MDSC-like monocytes/macrophages
S100A8
S100A9
FCN1
VCAN
HLA-DRB5
HLA-DQA1

Supplementary Figure 2 Single-cell transcriptomics of subclustered myeloid cells. a, Comparison of M1/M2 macrophage phenotypes across all macrophage clusters, calculated by the mean expressions of corresponding signature genes. Case, n = 9; controls, n = 4. The results are depicted in boxplots, in which the value for each patient is represented by a dot, the upper and lower bounds represent the 75% and 25% percentiles, respectively, the center bars indicate the medians, and the whiskers denote values up to 1.5 interquartile ranges above the 75% or below the 25% percentiles. b, Pseudotemporal transcriptional trajectory of monocytes/macrophages using DPT (calculated by Slingshot algorithms), PHATE and Monocle 2. c, t-SNE plots of detailed canonical marker genes of myeloid clusters. d, Canonical marker genes of myeloid-derived suppressor cell like monocytes/macrophages (MDSC-like monocytes/macrophages). MDSC-like monocytes/macrophages highly expressed S100A family genes of S100A8, S100A9, FCN1 and VCAN, and relatively low expressed of HLAs of HLA-DRB5 and HLA-DQA1. Source data are provided as a Source Data file.

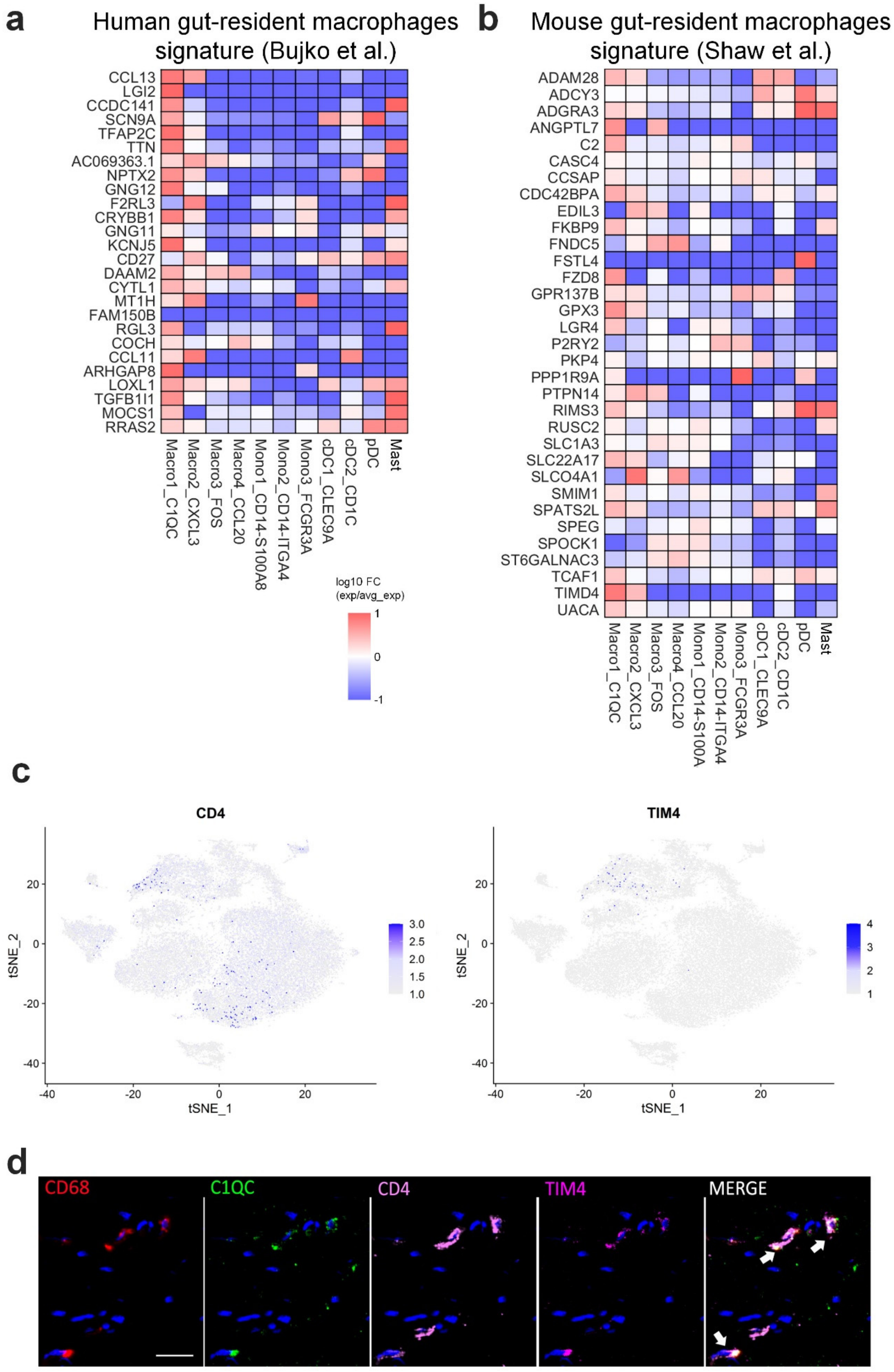
a
Human gut-resident macrophages signature (Bujko et al.)
CCL13
LGI2
CCDC141
SCN9A
TFAP2C
TTN
AC069363.1
NPTX2
GNG12
F2RL3
CRYBB1
GNG11
KCNJ5
CD27
DAAM2
CYTL1
MT1H
FAM150B
RGL3
COCH
CCL11
ARHGAP8
LOXL1
TGFB1I1
MOCS1
RRAS2
Macro1_C1QC
Macro2_CXCL3
Macro3_FOS
Macro4_CCL20
Mono1_CD14-S100A8
Mono2_CD14-ITGA4
Mono3_FCGR3A
cDC1_CLEC9A
cDC2_CD1C
pDC
Mast
log10 FC (exp/avg_exp)
1
0
-1
b
Mouse gut-resident macrophages signature (Shaw et al.)
ADAM28
ADCY3
ADGRA3
ANGPTL7
C2
CASC4
CCSAP
CDC42BPA
EDIL3
FKBP9
FNDC5
FSTL4
FZD8
GPR137B
GPX3
LGR4
P2RY2
PKP4
PPP1R9A
PTPN14
RIMS3
RUSC2
SLC1A3
SLC22A17
SLCO4A1
SMIM1
SPATS2L
SPEG
SPOCK1
ST6GALNAC3
TCAF1
TIMD4
UACA
Macro1_C1QC
Macro2_CXCL3
Macro3_FOS
Macro4_CCL20
Mono1_CD14-S100A
Mono2_CD14-ITGA4
Mono3_FCGR3A
cDC1_CLEC9A
cDC2_CD1C
pDC
Mast
c
CD4
TIM4
tSNE_2
tSNE_1
20
0
-20
-40
-40
-20
0
20
3.0
2.5
2.0
1.5
1.0
4
3
2
1
d
CD68
C1QC
CD4
TIM4
MERGE

Supplementary Figure 3 C1QC⁺ macrophages expressed transcriptional gene set and marker genes similar to those of previously reported gut-resident macrophages. a, b, C1QC⁺ macrophages expressed transcriptional gene set similar to those of previously reported gut-resident macrophages of human (c) and mouse (c). c, d, t-SNE plots showed the high expressions of *CD4* and *TIM4* in C1QC⁺ macrophages (c) and validated by multicolor IHC (d). CD68 (red), C1QC (green), CD4 (pink), TIM4 (magenta). Scale bar, 20 μm. Arrow, C1QC⁺ macrophages. Source data are provided as a Source Data file.

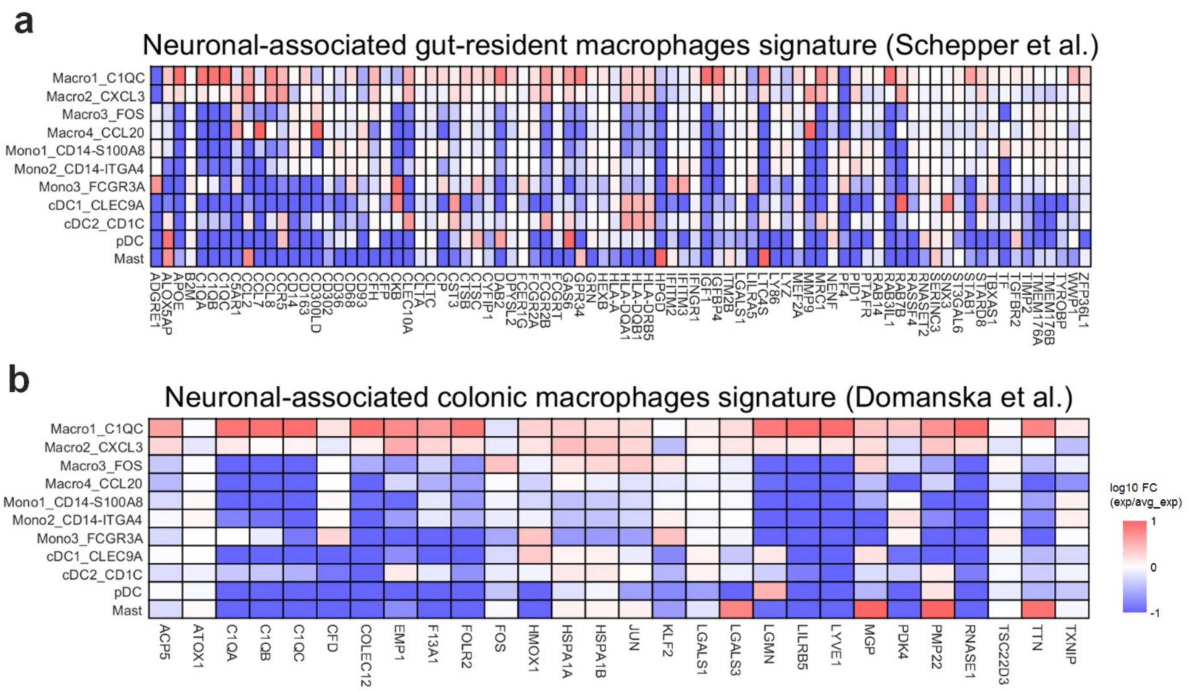

Supplementary Figure 4 Comparison of marker genes of myeloid cells with previously reported neuronal-associated gut-resident macrophages gene sets. a, Comparison of marker genes of myeloid clusters with previously reported neuronal-associated gut-resident macrophages gene set. b, Comparison of marker genes of myeloid cells with previously reported neuronal-associated colonic macrophages gene set. In the study of Domanska, they performed scRNA-seq and found neuronal-associated colonic macrophages were similar to gut-resident macrophages. Source data are provided as a Source Data file.

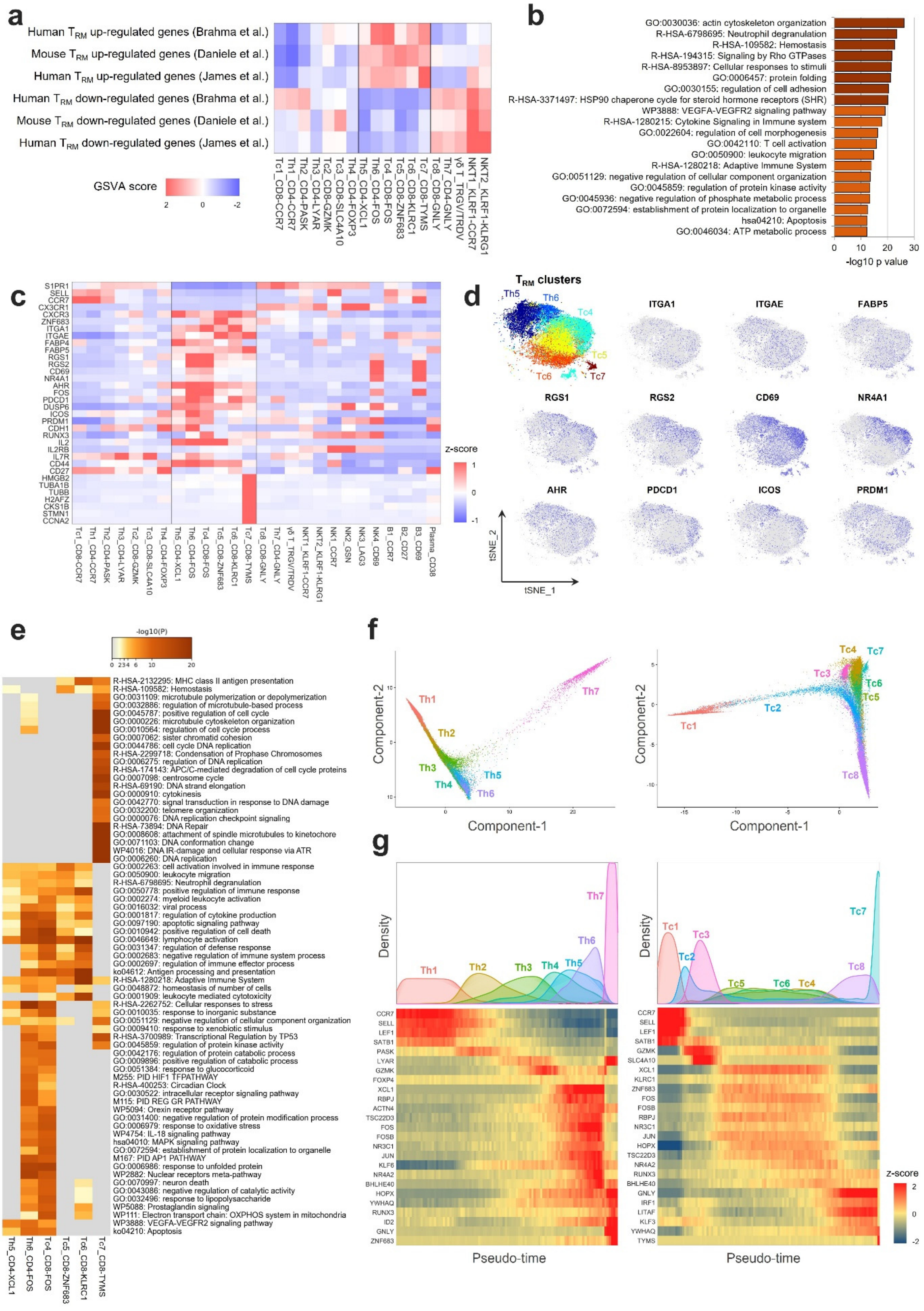
a
Human TRM up-regulated genes (Brahma et al.)
Mouse TRM up-regulated genes (Daniele et al.)
Human TRM up-regulated genes (James et al.)
Human TRM down-regulated genes (Brahma et al.)
Mouse TRM down-regulated genes (Daniele et al.)
Human TRM down-regulated genes (James et al.)
GSVA score
2
0
-2
Tc1_CD8-CCR7
Th1_CD4-CCR7
Th2_CD4-PASK
Th3_CD4-LYAR
Tc2_CD8-GZMK
Tc3_CD8-SLC4A10
Th4_CD4-FOXP3
Th5_CD4-XCL1
Th6_CD4-FOS
Tc4_CD8-FOS
Tc5_CD8-ZNF683
Tc6_CD8-KLRC1
Tc7_CD8-TYMS
Tc8_CD8-GNLY
Th7_CD4-GNLY
γδ T_TRGV/TRDV
NKT1_KLRF1-CCR7
NKT2_KLRF1-KLRG1
b
GO:0030036: actin cytoskeleton organization
R-HSA-6798695: Neutrophil degranulation
R-HSA-109582: Hemostasis
R-HSA-194315: Signaling by Rho GTPases
R-HSA-8953897: Cellular responses to stimuli
GO:0006457: protein folding
GO:0030155: regulation of cell adhesion
R-HSA-3371497: HSP90 chaperone cycle for steroid hormone receptors (SHR)
WP3888: VEGFA-VEGFR2 signaling pathway
R-HSA-1280215: Cytokine Signaling in Immune system
GO:0022604: regulation of cell morphogenesis
GO:0042110: T cell activation
GO:0050900: leukocyte migration
R-HSA-1280218: Adaptive Immune System
GO:0051129: negative regulation of cellular component organization
GO:0045859: regulation of protein kinase activity
GO:0045936: negative regulation of phosphate metabolic process
GO:0072594: establishment of protein localization to organelle
hsa04210: Apoptosis
GO:0046034: ATP metabolic process
0
10
20
30
-log10 p value
c
S1PR1
SELL
CCR7
CX3CR1
CXCR3
ZNF683
ITGA1
ITGAE
FABP4
FABP5
RGS1
RGS2
CD69
NR4A1
AHR
FOS
PDCD1
DUSP6
ICOS
PRDM1
CDH1
RUNX3
IL2
IL2RB
IL7R
CD44
CD27
HMGB2
TUBA1B
TUBB
H2AFZ
CKS1B
STMN1
CCNA2
z-score
1
0
-1
Tc1_CD8-CCR7
Th1_CD4-CCR7
Th2_CD4-PASK
Th3_CD4-LYAR
Tc2_CD8-GZMK
Tc3_CD8-SLC4A10
Th4_CD4-FOXP3
Th5_CD4-XCL1
Th6_CD4-FOS
Tc4_CD8-FOS
Tc5_CD8-ZNF683
Tc6_CD8-KLRC1
Tc7_CD8-TYMS
Tc8_CD8-GNLY
Th7_CD4-GNLY
γδ T_TRGV/TRDV
NKT1_KLRF1-CCR7
NKT2_KLRF1-KLRG1
NK1_CCR7
NK2_GSN
NK3_LAG3
NK4_CD69
B1_CCR7
B2_CD27
B3_CD69
Plasma_CD38
d
TRM clusters
Th5
Th6
Tc4
Tc5
Tc6
Tc7
ITGA1
ITGAE
FABP5
RGS1
RGS2
CD69
NR4A1
AHR
PDCD1
ICOS
PRDM1
tSNE_2
tSNE_1
e
-log10(P)
0 2 3 4 6 10 20
R-HSA-2132295: MHC class II antigen presentation
R-HSA-109582: Hemostasis
GO:0031109: microtubule polymerization or depolymerization
GO:0032886: regulation of microtubule-based process
GO:0045787: positive regulation of cell cycle
GO:0000226: microtubule cytoskeleton organization
GO:0010564: regulation of cell cycle process
GO:0007062: sister chromatid cohesion
GO:0044786: cell cycle DNA replication
R-HSA-2299718: Condensation of Prophase Chromosomes
GO:0006275: regulation of DNA replication
R-HSA-174143: APC/C-mediated degradation of cell cycle proteins
GO:0007098: centrosome cycle
R-HSA-69190: DNA strand elongation
GO:0000910: cytokinesis
GO:0042770: signal transduction in response to DNA damage
GO:0032200: telomere organization
GO:0000076: DNA replication checkpoint signaling
R-HSA-73894: DNA Repair
GO:0008608: attachment of spindle microtubules to kinetochore
GO:0071103: DNA conformation change
WP4016: DNA IR-damage and cellular response via ATR
GO:0006260: DNA replication
GO:0002263: cell activation involved in immune response
GO:0050900: leukocyte migration
R-HSA-6798695: Neutrophil degranulation
GO:0050778: positive regulation of immune response
GO:0002274: myeloid leukocyte activation
GO:0016032: viral process
GO:0001817: regulation of cytokine production
GO:0097190: apoptotic signaling pathway
GO:0010942: positive regulation of cell death
GO:0046649: lymphocyte activation
GO:0031347: regulation of defense response
GO:0002683: negative regulation of immune system process
GO:0002697: regulation of immune effector process
ko04612: Antigen processing and presentation
R-HSA-1280218: Adaptive Immune System
GO:0048872: homeostasis of number of cells
GO:0001909: leukocyte mediated cytotoxicity
R-HSA-2262752: Cellular responses to stress
GO:0010035: response to inorganic substance
GO:0051129: negative regulation of cellular component organization
GO:0009410: response to xenobiotic stimulus
R-HSA-3700989: Transcriptional Regulation by TP53
GO:0045859: regulation of protein kinase activity
GO:0042176: regulation of protein catabolic process
GO:0009896: positive regulation of catabolic process
GO:0051384: response to glucocorticoid
M255: PID HIF1 TFPATHWAY
R-HSA-400253: Circadian Clock
GO:0030522: intracellular receptor signaling pathway
M115: PID REG GR PATHWAY
WP5094: Orexin receptor pathway
GO:0031400: negative regulation of protein modification process
GO:0006979: response to oxidative stress
WP4754: IL-18 signaling pathway
hsa04010: MAPK signaling pathway
GO:0072594: establishment of protein localization to organelle
M167: PID AP1 PATHWAY
GO:0006986: response to unfolded protein
WP2882: Nuclear receptors meta-pathway
GO:0070997: neuron death
GO:0043086: negative regulation of catalytic activity
GO:0032496: response to lipopolysaccharide
WP5088: Prostaglandin signaling
WP111: Electron transport chain: OXPHOS system in mitochondria
WP3888: VEGFA-VEGFR2 signaling pathway
ko04210: Apoptosis
Th5_CD4-XCL1
Th6_CD4-FOS
Tc4_CD8-FOS
Tc5_CD8-ZNF683
Tc6_CD8-KLRC1
Tc7_CD8-TYMS
f
Th1
Th2
Th3
Th4
Th5
Th6
Th7
Component-2
Component-1
Tc1
Tc2
Tc3
Tc4
Tc5
Tc6
Tc7
Tc8
Component-2
Component-1
g
Density
Th1
Th2
Th3
Th4
Th5
Th6
Th7
CCR7
SELL
LEF1
SATB1
PASK
LYAR
GZMK
FOXP4
XCL1
RBPJ
ACTN4
TSC22D3
FOS
FOSB
NR3C1
JUN
KLF6
NR4A2
BHLHE40
HOPX
YWHAQ
RUNX3
ID2
GNLY
ZNF683
Pseudo-time
Density
Tc1
Tc2
Tc3
Tc5
Tc6
Tc4
Tc7
Tc8
CCR7
SELL
LEF1
SATB1
GZMK
SLC4A10
XCL1
KLRC1
ZNF683
FOS
FOSB
RBPJ
NR3C1
JUN
HOPX
TSC22D3
NR4A2
RUNX3
BHLHE40
GNLY
IRF1
LITAF
KLF3
YWHAQ
TYMS
z-score
2
0
-2
Pseudo-time

Supplementary Figure 5 Single-cell transcriptomics of T_{RM} subclusters. a, Gene set variation analysis (GSVA) analysis comparing the marker genes for T_{RM} with previously reported signatures of T_{RM}. b, Metascape analysis of merged marker genes of T_{RM}. c, d, Heatmap (c) and t-SNE plots (d) of relative expression levels of canonical marker genes across T_{RM}. e, Metascape analysis of marker genes of T_{RM} subclusters. f, Pseudotemporal trajectories of $CD4^+$ and $CD8^+$ T cells using the DPT algorithm. g, Heatmap showing the continuous phenotypic variations and pseudotime with cell-type-specific gene changes. Statistics: *P* value in (b, e): Fisher's exact test. Source data are provided as a Source Data file.

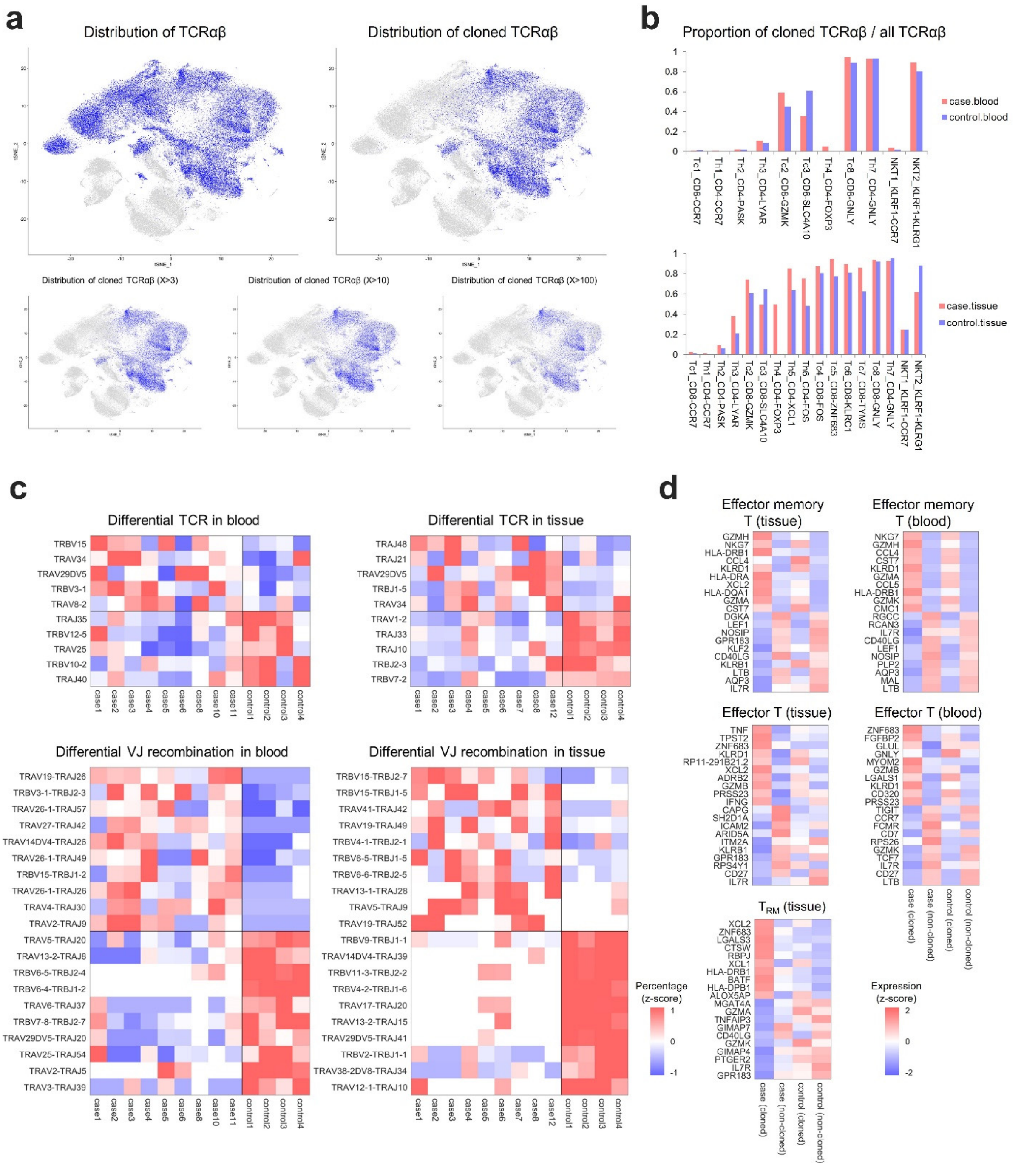

Supplementary Figure 6 Detailed TCR repertoire based on T cell subclusters in achalasia and controls. a, t-SNE plots of the TCR repertoire. b, Comparison of the cloned TCR repertoire of different T cells between achalasia and controls. c, Heatmap showing differential TCR and VJ recombinations between achalasia and controls. d, Comparison of DEGs associated with cloned

expanded and unexpanded T cells between achalasia and controls. Source data are provided as a Source Data file.

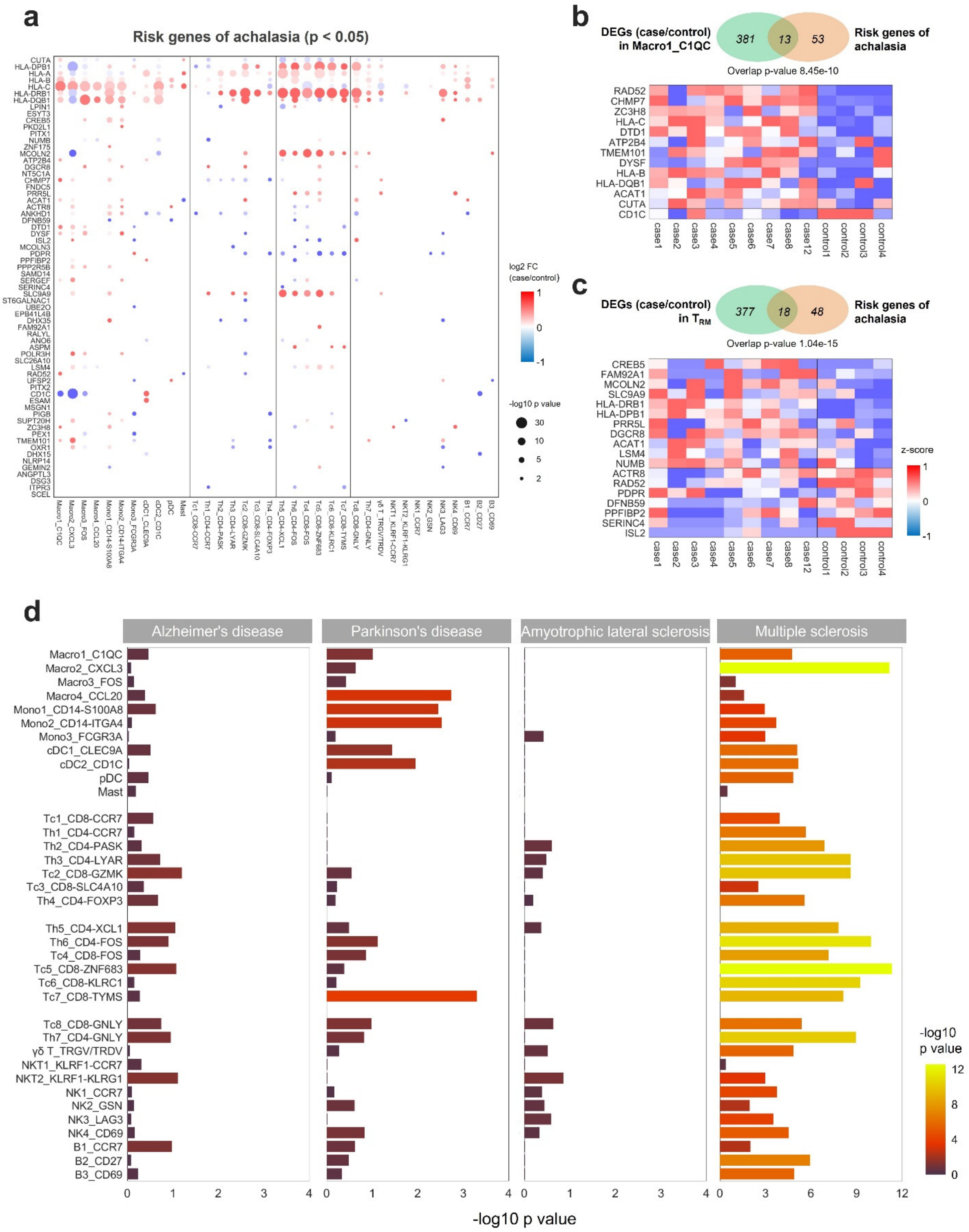
a
Risk genes of achalasia (p < 0.05)
log2 FC (case/control)
1
0
-1
-log10 p value
30
10
5
2
b
DEGs (case/control) in Macro1_C1QC
381
13
53
Risk genes of achalasia
Overlap p-value 8.45e-10
RAD52
CHMP7
ZC3H8
HLA-C
DTD1
ATP2B4
TMEM101
DYSF
HLA-B
HLA-DQB1
ACAT1
CUTA
CD1C
case1
case2
case3
case4
case5
case6
case7
case8
case12
control1
control2
control3
control4
c
DEGs (case/control) in TRM
377
18
48
Risk genes of achalasia
Overlap p-value 1.04e-15
CREB5
FAM92A1
MCOLN2
SLC9A9
HLA-DRB1
HLA-DPB1
PRR5L
DGCR8
ACAT1
LSM4
NUMB
ACTR8
RAD52
PDPR
DFNB59
PPFIBP2
SERINC4
ISL2
z-score
1
0
-1
case1
case2
case3
case4
case5
case6
case7
case8
case12
control1
control2
control3
control4
d
Alzheimer's disease
Parkinson's disease
Amyotrophic lateral sclerosis
Multiple sclerosis
Macro1_C1QC
Macro2_CXCL3
Macro3_FOS
Macro4_CCL20
Mono1_CD14-S100A8
Mono2_CD14-ITGA4
Mono3_FCGR3A
cDC1_CLEC9A
cDC2_CD1C
pDC
Mast
Tc1_CD8-CCR7
Th1_CD4-CCR7
Th2_CD4-PASK
Th3_CD4-LYAR
Tc2_CD8-GZMK
Tc3_CD8-SLC4A10
Th4_CD4-FOXP3
Th5_CD4-XCL1
Th6_CD4-FOS
Tc4_CD8-FOS
Tc5_CD8-ZNF683
Tc6_CD8-KLRC1
Tc7_CD8-TYMS
Tc8_CD8-GNLY
Th7_CD4-GNLY
γδ T_TRGV/TRDV
NKT1_KLRF1-CCR7
NKT2_KLRF1-KLRG1
NK1_CCR7
NK2_GSN
NK3_LAG3
NK4_CD69
B1_CCR7
B2_CD27
B3_CD69
0 1 2 3 4
0 1 2 3 4
0 1 2 3 4
0 3 6 9 12
-log10 p value
-log10 p value
12
8
4
0

Supplementary Figure 7 DEGs for C1QC$^+$ macrophages and T_{RM} overlapped with risk genes for achalasia and other neurodegenerative diseases. a, Comparison of DEGs for myeloid cells and T_{RM} with risk genes for achalasia identified by whole-exome sequencing (WES). Only the points with $P < 0.05$ were illustrated. b, c, Specific DEGs for C1QC$^+$ macrophages (b) and T_{RM} (c) overlapped with risk genes for achalasia identified by WES. d, Significant levels of the overlap between DEGs of achalasia and risk genes for other neurodegenerative diseases from the GWAS catalog (https://www.ebi.ac.uk/gwas/)), including PD, AD, ALS, and MS. Both the axis and heat key are represented as p value (Fisher's exact test). Statistics: *P* value in (a): two-sided Wilcoxon rank sum test. *P* value in (b-d): Fisher's exact test.

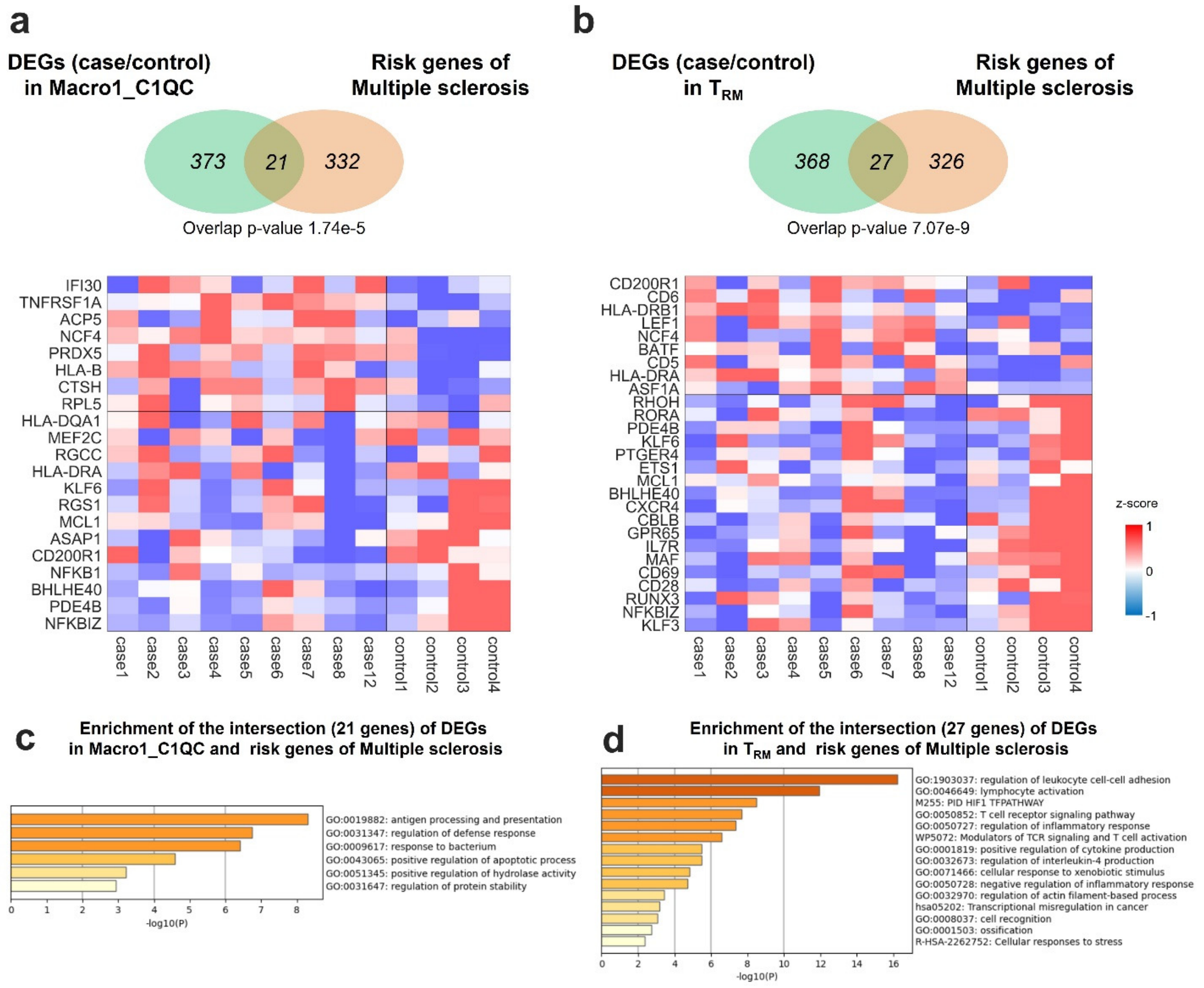

Supplementary Figure 8 DEGs for C1QC$^+$ macrophages and T$_{RM}$ overlapped with risk genes for MS. a, Venn diagram (upper) and heatmap (lower) comparing the DEGs for C1QC$^+$ macrophages with risk genes for MS identified by GWAS. b, Venn diagram (upper) and heatmap (lower) comparing the DEGs for T$_{RM}$ with risk genes for MS identified by GWAS. c, d, Metascape analysis of overlapped DEGs in C1QC$^+$ macrophages (c) and T$_{RM}$ (d) with risk genes for MS identified by GWAS. Statistics: *P* value in (a-d): Fisher's exact test. DEGs, differentially expressed genes. Source data are provided as a Source Data file.

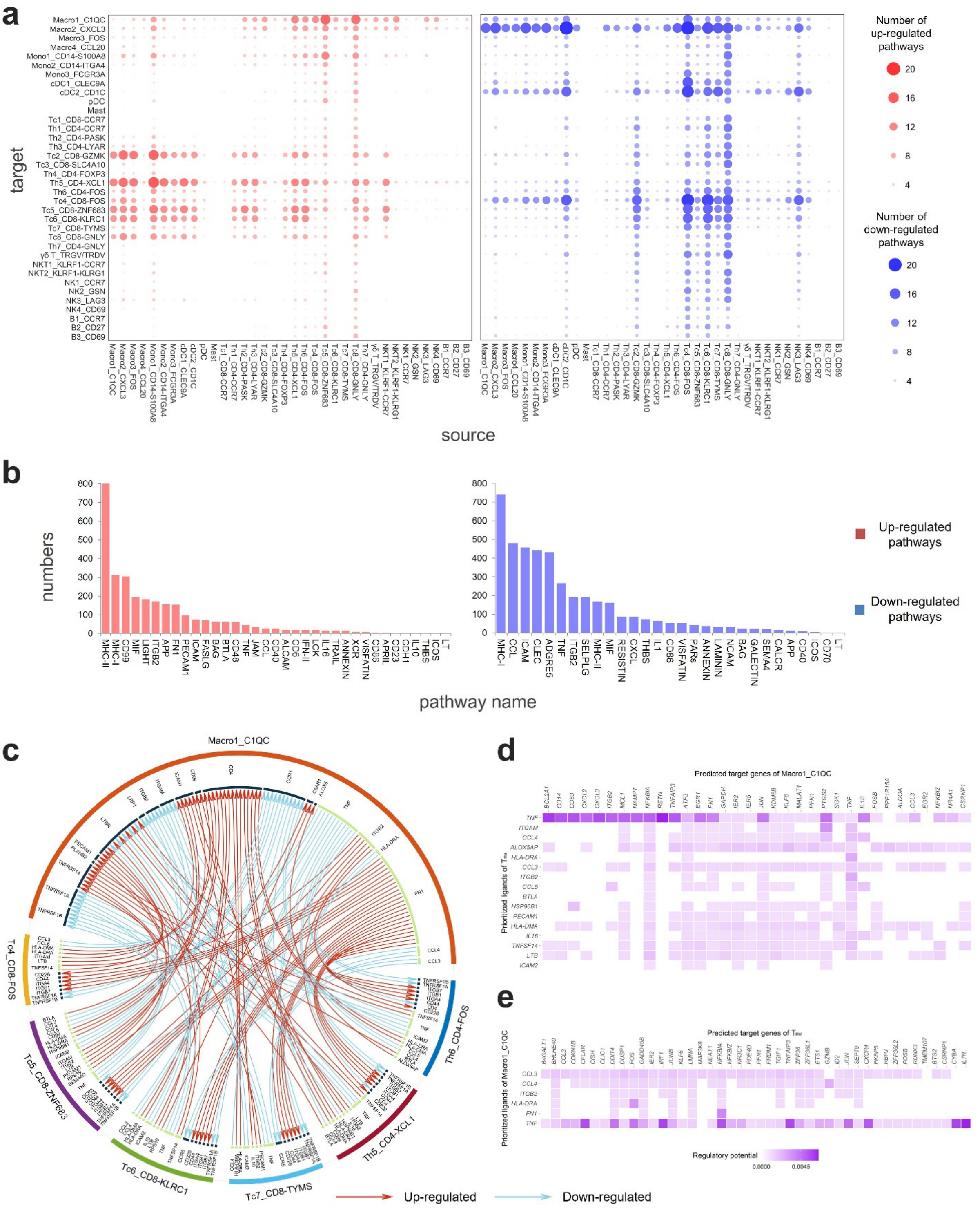

a
target
source
Number of up-regulated pathways
20
16
12
8
4
Number of down-regulated pathways
20
16
12
8
4
Macro1_C1QC
Macro2_CXCL3
Macro3_FOS
Macro4_CCL20
Mono1_CD14-S100A8
Mono2_CD14-ITGA4
Mono3_FCGR3A
cDC1_CLEC9A
cDC2_CD1C
pDC
Mast
Tc1_CD8-CCR7
Th1_CD4-CCR7
Th2_CD4-PASK
Th3_CD4-LYAR
Tc2_CD8-GZMK
Tc3_CD8-SLC4A10
Th4_CD4-FOXP3
Th5_CD4-XCL1
Th6_CD4-FOS
Tc4_CD8-FOS
Tc5_CD8-ZNF683
Tc6_CD8-KLRC1
Tc7_CD8-TYMS
Tc8_CD8-GNLY
Th7_CD4-GNLY
γδ T_TRGV/TRDV
NKT1_KLRF1-CCR7
NKT2_KLRF1-KLRG1
NK1_CCR7
NK2_GSN
NK3_LAG3
NK4_CD69
B1_CCR7
B2_CD27
B3_CD69
b
numbers
pathway name
Up-regulated pathways
Down-regulated pathways
MHC-II
MHC-I
CD99
MIF
LIGHT
ITGB2
APP
FN1
PECAM1
ICAM
FASLG
BAG
BTLA
CD48
TNF
JAM
CCL
CD40
ALCAM
CD6
IFN-II
LCK
IL16
TRAIL
ANNEXIN
XCR
VISFATIN
CD86
APRIL
CD23
CDH1
IL10
THBS
ICOS
LT
CLEC
ADGRE5
SELPLG
RESISTIN
CXCL
IL1
PARs
LAMININ
NCAM
GALECTIN
SEMA4
CALCR
CD70
c
Macro1_C1QC
Tc4_CD8-FOS
Tc5_CD8-ZNF683
Tc6_CD8-KLRC1
Tc7_CD8-TYMS
Th5_CD4-XCL1
Th6_CD4-FOS
Up-regulated
Down-regulated
d
Predicted target genes of Macro1_C1QC
Prioritized ligands of T_{RM}
e
Predicted target genes of T_{RM}
Prioritized ligands of Macro1_C1QC
Regulatory potential
0.0000
0.0045

Supplementary Figure 9 Achalasia was characterized by altered interactions between C1QC$^+$ macrophages and T$_{RM}$. a, Heatmap of the putative differential receptor-ligand interactions in achalasia compared with controls. b, Specific upregulated and downregulated interactions in achalasia compared with controls. c, Circos plot showing all putative upregulated or downregulated interactions between C1QC$^+$ macrophages and T$_{RM}$ subclusters for achalasia. d, e, Top-predicted differentially expressed ligands and target genes between C1QC$^+$ macrophages and T$_{RM}$. Source data are provided as a Source Data file.

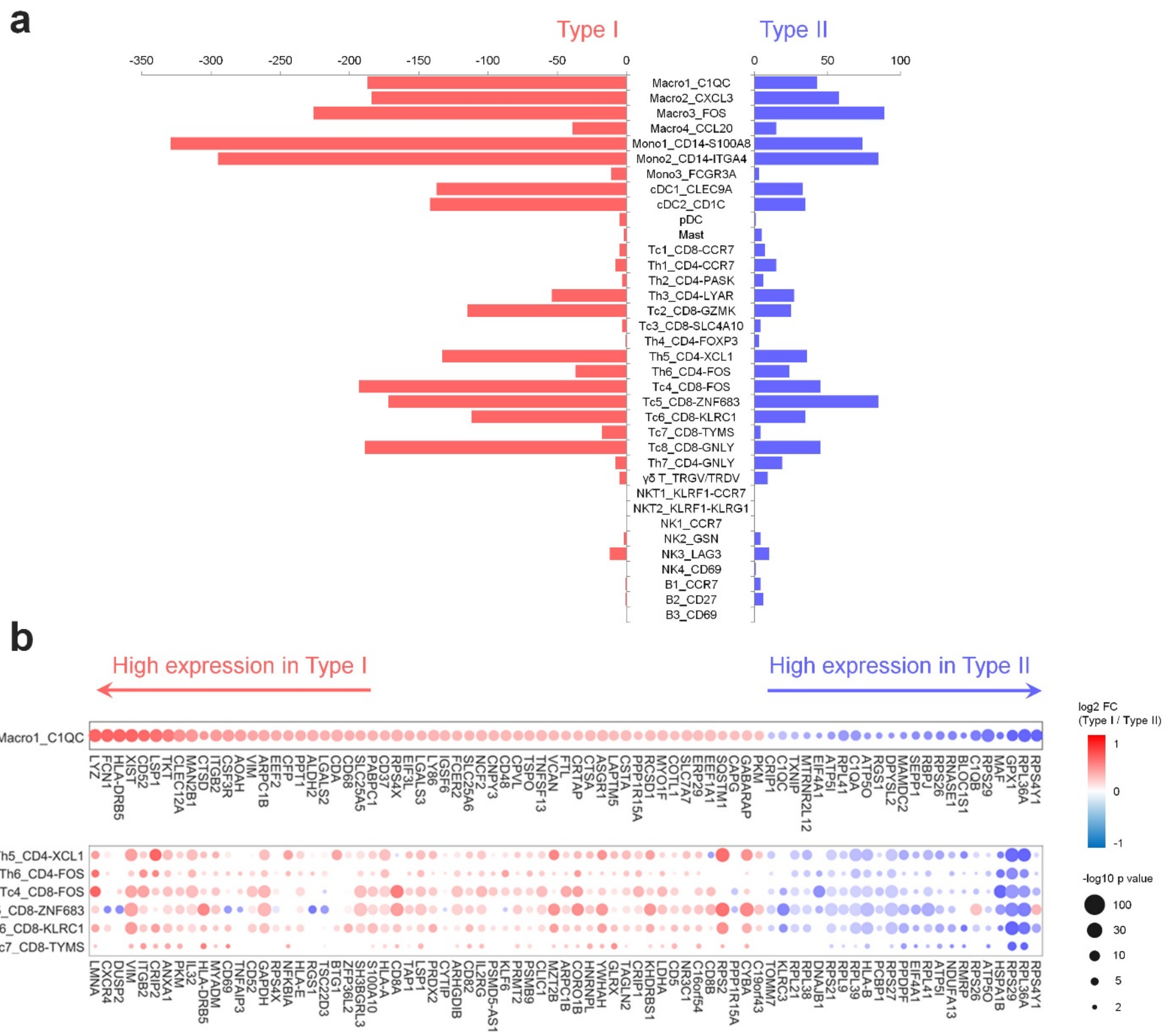

Supplementary Figure 10 DEGs in type I and type II achalasia. a, Bar graph showing the upregulated DEGs in myeloid cells and lymphocytes in type I and type II achalasia, respectively. b, Dot plot showing the expression patterns for selected DEGs in C1QC^{+} macrophages and T$_{RM}$ in type I and type II achalasia. Statistics: *P* value in (b): two-sided Wilcoxon rank sum test. Source data are provided as a Source Data file.

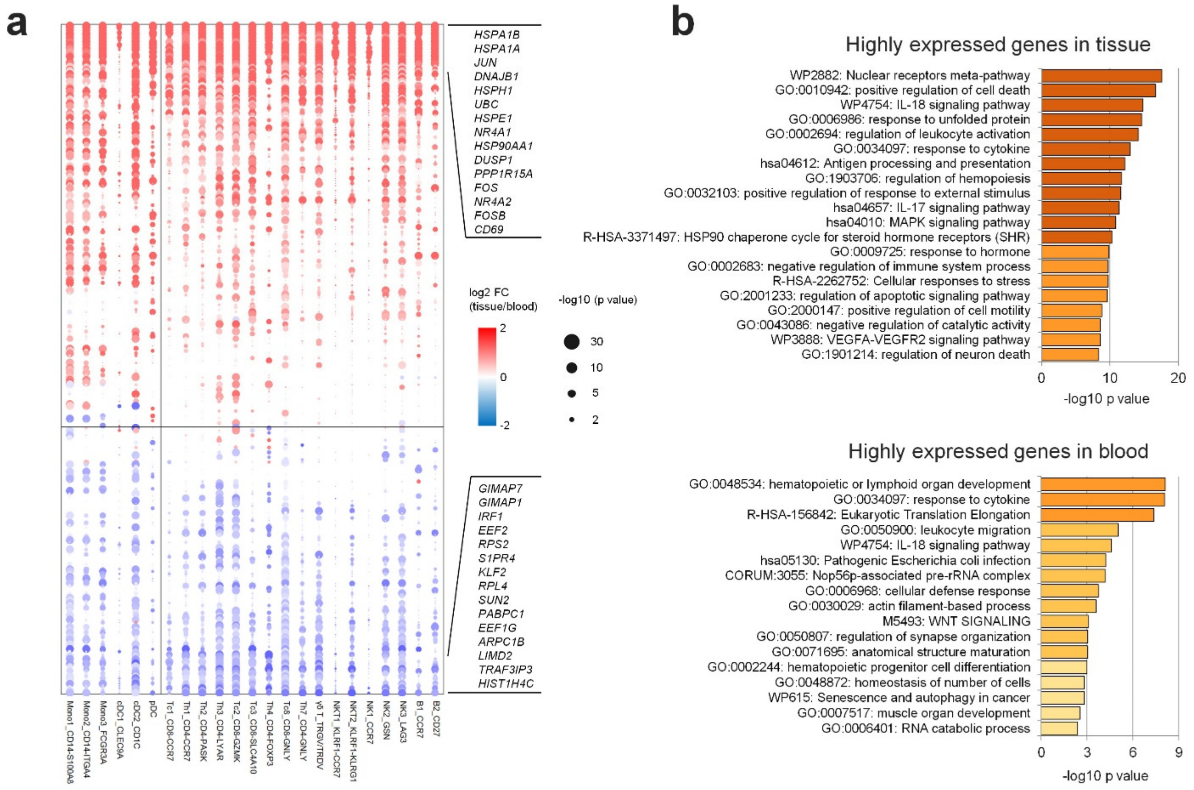

Supplementary Figure 11 Transcriptional difference of immune cells between peripheral blood and LES tissue. a, Dot plot showing differential expressed genes for immune cells between peripheral blood and LES tissue. Statistics: two-sided Wilcoxon rank sum test. b, Metascape analysis of merged differential expressed genes between peripheral blood and LES tissue. Statistics: Fisher's exact test. Source data are provided as a Source Data file.

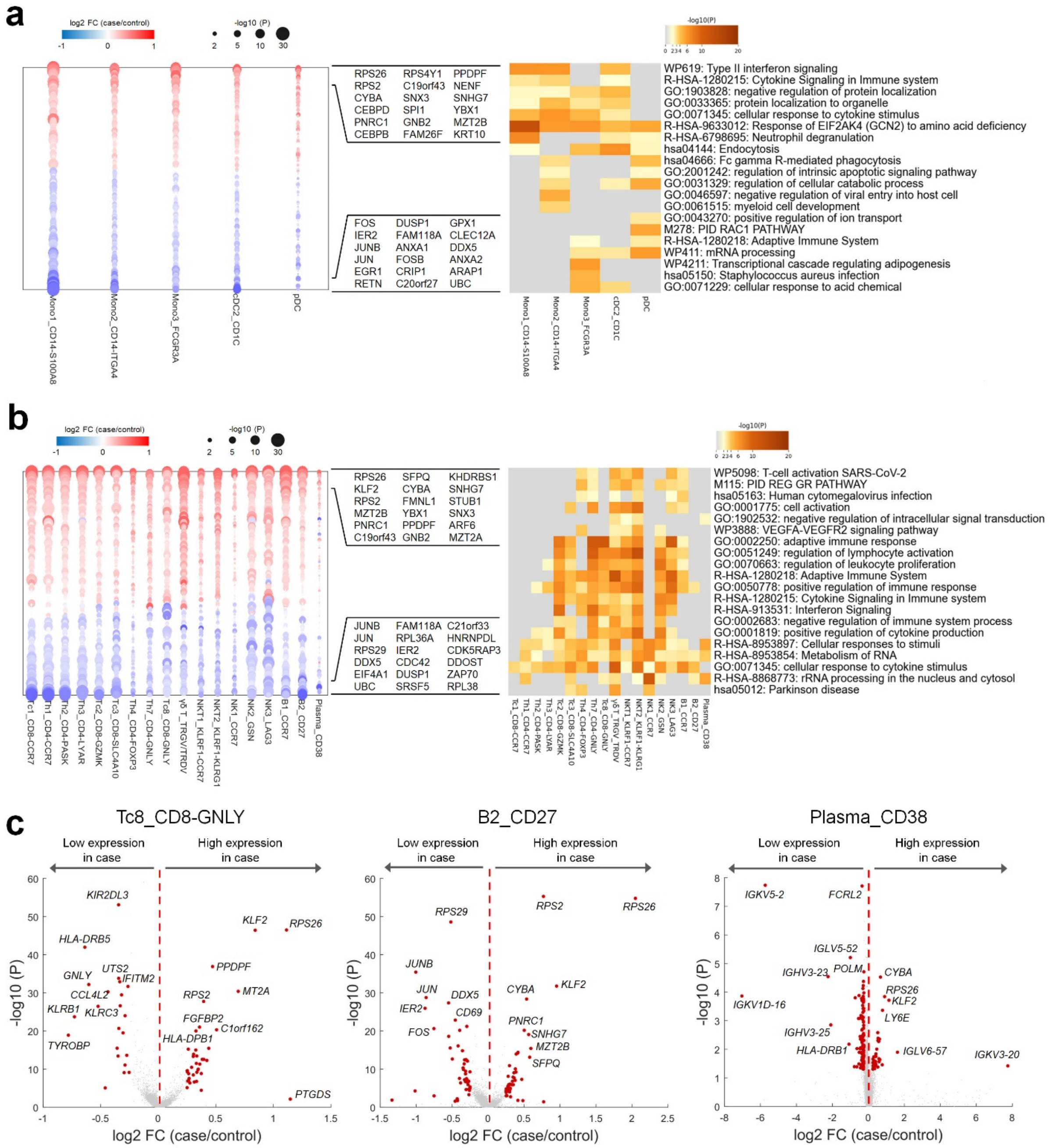

Supplementary Figure 12 Achalasia-associated transcriptional changes of immune cells in peripheral blood. a, Dot plot (left) and Metascape analysis (right) of DEGs for peripheral blood myeloid cells between achalasia and controls. b, Dot plot (left) and Metascape analysis (right) of DEGs for peripheral blood lymphocytes between achalasia and controls. c, Volcano plots showing the DEGs of GNLY^{+} CD8^{+} T cells, CD27^{+} B cells and plasma in peripheral blood between achalasia and controls. Statistics: *P* value in Dot plots (a-left, b-left) and Volcano plots (c): two-

sided Wilcoxon rank sum test; *P* value in Metascape analysis (a-right, b-right): Fisher's exact test. Source data are provided as a Source Data file.

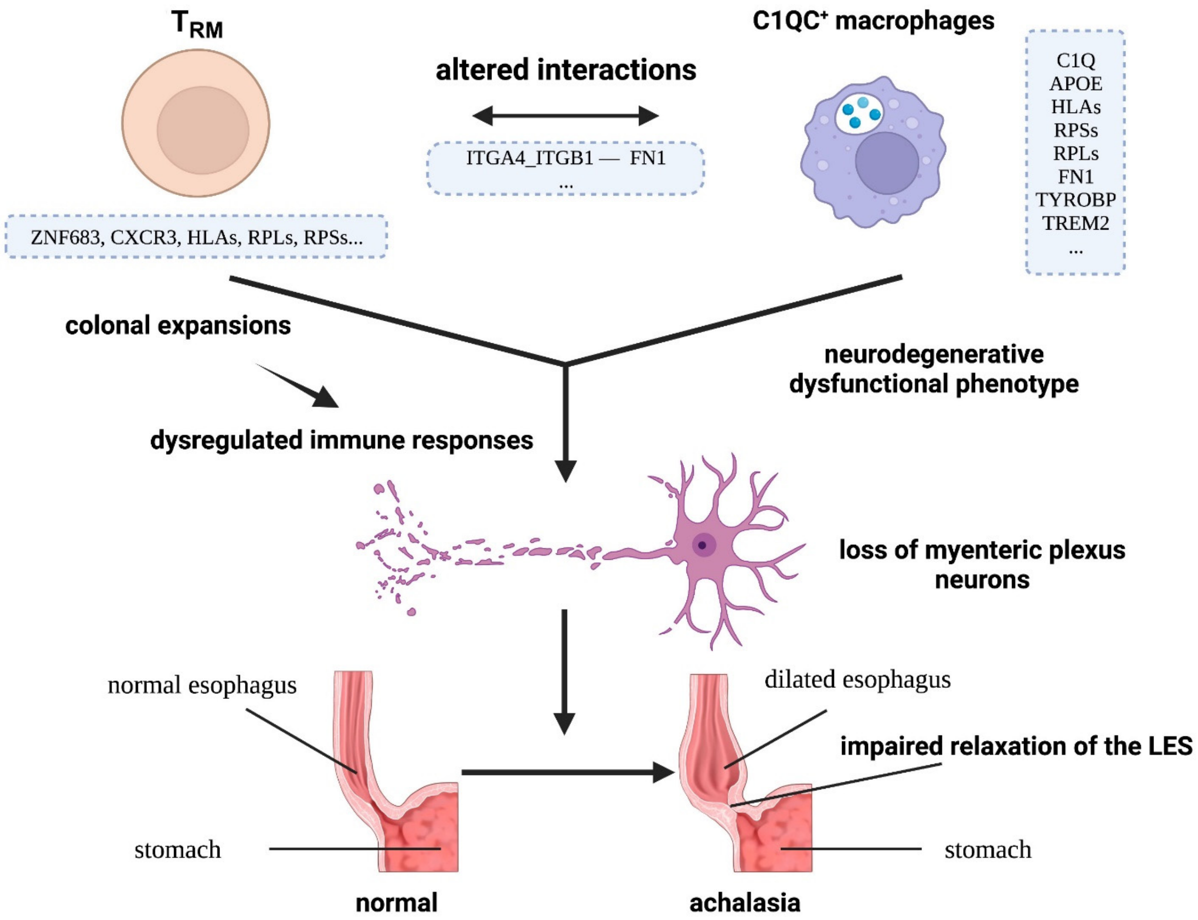

Supplementary Figure 13 Illustration showing the possible pathophysiology of achalasia. C1QC$^+$ macrophages and TRM were significantly expanded and localized surrounding the myenteric plexus in the LES tissue of achalasia. C1QC$^+$ macrophages were transcriptionally similar to microglia of the CNS and exhibited a neurodegenerative dysfunctional phenotype in achalasia. TRM also expressed transcripts of dysregulated immune responses in achalasia. By potential interactions, the C1QC$^+$ macrophages together with TRM might cause the ganglionitis and loss of myenteric neurons. The loss of myenteric neurons could lead to the aberrant esophageal peristalsis, dilated esophagus, and impaired relaxation of the LES, and finally the occurrence of achalasia.
