# Supplementary material for: A single-cell transcriptional landscape of immune cells shows disease-specific changes of T cell and macrophage populations in human achalasia
Source: Nat Commun. 2023 Aug 4;14:4685. doi: 10.1038/s41467-023-39750-5 (PMC10403544; doi:10.1038/s41467-023-39750-5)
Supplement: Supplementary file 3 — Description of Additional Supplementary Files [file 41467_2023_39750_MOESM3_ESM.pdf]

Description of Additional Supplementary Files

Supplementary Data 1. Sample and data information.

Supplementary Data 2. Marker genes for annotations.

Supplementary Data 3. cyTOF analysis.

Supplementary Data 4. Subclustered myeloid cells.

Supplementary Data 5. $C1QC^{+}$ macrophages

Supplementary Data 6. Subclustered lymphocytes.

Supplementary Data 7. TCR analysis.

Supplementary Data 8. BCR analysis.

Supplementary Data 9. Risk genes.

Supplementary Data 10. Cell-cell communication.

Supplementary Data 11. Subtype analysis.

Supplementary Data 12. Comparison between blood and tissue.

Supplementary Data 13. Antibody list.
