# Supplementary material for: A single-cell transcriptional landscape of immune cells shows disease-specific changes of T cell and macrophage populations in human achalasia
Source: Nat Commun. 2023 Aug 4;14:4685. doi: 10.1038/s41467-023-39750-5 (PMC10403544; doi:10.1038/s41467-023-39750-5)
Supplement: Supplementary file 5 — Reporting Summary [file 41467_2023_39750_MOESM5_ESM.pdf]

nature portfolio

Corresponding author(s): Quanlin Li, Pinghong Zhou, Lunan Chen

Last updated by author(s): Apr 9, 2023

Reporting Summary

Nature Portfolio wishes to improve the reproducibility of the work that we publish. This form provides structure for consistency and transparency in reporting. For further information on Nature Portfolio policies, see our Editorial Policies and the Editorial Policy Checklist.

Statistics

For all statistical analyses, confirm that the following items are present in the figure legend, table legend, main text, or Methods section.

n/a	Confirmed	
☐	☒	The exact sample size (*n*) for each experimental group/condition, given as a discrete number and unit of measurement
☐	☒	A statement on whether measurements were taken from distinct samples or whether the same sample was measured repeatedly
☐	☒	The statistical test(s) used AND whether they are one- or two-sided<br>*Only common tests should be described solely by name; describe more complex techniques in the Methods section.*
☐	☒	A description of all covariates tested
☐	☒	A description of any assumptions or corrections, such as tests of normality and adjustment for multiple comparisons
☐	☒	A full description of the statistical parameters including central tendency (e.g. means) or other basic estimates (e.g. regression coefficient) AND variation (e.g. standard deviation) or associated estimates of uncertainty (e.g. confidence intervals)
☐	☒	For null hypothesis testing, the test statistic (e.g. *F*, *t*, *r*) with confidence intervals, effect sizes, degrees of freedom and *P* value noted<br>*Give P values as exact values whenever suitable.*
☒	☐	For Bayesian analysis, information on the choice of priors and Markov chain Monte Carlo settings
☒	☐	For hierarchical and complex designs, identification of the appropriate level for tests and full reporting of outcomes
☒	☐	Estimates of effect sizes (e.g. Cohen's *d*, Pearson's *r*), indicating how they were calculated

Our web collection on statistics for biologists contains articles on many of the points above.

Software and code

Policy information about availability of computer code

Data collection	For single-cell RNA-seq, TCR-seq and BCR-seq, single-cell suspensions were loaded on the Chromium System using the Chromium Single Cell 5' Library & Gel Bead Kit v3 (10X Genomics). Following capture and lysis, single-cell gene expression and libraries were constructed according to the manufacturer's instructions. Completed libraries were sequenced on NovaSeq6000 (Illumina) platforms using 2 × 150 chemistry at a targeted median read depth of about 63,000 reads per cell from total gene expression libraries and about 13,000 reads per cell for TCR libraries (cycle specifications 150:8:0:150 [R1:i7:i5:R2]). Illumina basecall files (*.bcl) were converted to fastqs using the CellRanger v3.0.1 (10X Genomics) pipeline with recommended parameters.<br>Multicolor IHC imaging was collected by Pannoramic Digital Slide Scanners and analyzed by Pannoramic Scanner (Akoya Biosciences).
Data analysis	For single-cell RNA-seq, reads were mapped by CellRanger v3.0.1 onto human reference genome GRCh38. For single-cell TCR-seq and BCR-seq, reads were mapped by CellRanger v3.0.1 onto human VDJ reference genome (GRCh38-alts-ensembl). Downstream bioinformatics analyses were performed on R package: Seurat (v4.0.2), batchelor (v1.6.2), clusterProfiler (v3.18.1), org.Hs.eg.db (v3.14), monocle (v2.4.0), phateR (v1.0.7), slingshot (v2.4.0), GSVA (v1.38.2), CellChat (v1.1.0), nichenetr (v1.1.0); MATLAB package: Diffusion pseudotime (v1.0); Metascape (v3.5) (https://metascape.org).<br>Mass spectrometry analysis was performed on a TIMS-TOF Pro mass spectrometer (Bruker Daltonics). Tandem mass spectra were analyzed using PEAKS Online (Bioinformatics Solutions Inc).<br>CyTOF data was analyzed using FlowJo Version 10.8.1.<br>Quantifications of Multicolor IHC and CyTOF data were analyzed using GraphPad Prism (v8).

For manuscripts utilizing custom algorithms or software that are central to the research but not yet described in published literature, software must be made available to editors and reviewers. We strongly encourage code deposition in a community repository (e.g. GitHub). See the Nature Portfolio guidelines for submitting code & software for further information.

Data

Policy information about availability of data

All manuscripts must include a data availability statement. This statement should provide the following information, where applicable:

- Accession codes, unique identifiers, or web links for publicly available datasets
- A description of any restrictions on data availability
- For clinical datasets or third party data, please ensure that the statement adheres to our policy

Single-cell RNA-seq, TCR-seq, BCR-seq data generated in this study have been deposited in the Genome Sequence Archive for Human (GSA-Human) database of National Genomics Data Center, under the code HRA002791 and shared URL: https://ngdc.cncb.ac.cn/gsa-human/s/iIL1CjMX. There are no restrictions on data availability. All other relevant data supporting the findings of this study are available within the article and its Supplementary Information or Source Data files.

Human research participants

Policy information about studies involving human research participants and Sex and Gender in Research.

Reporting on sex and gender	The cases consisted of 6 males and 5 females. The controls consisted of 2 males and 2 females. Detailed information of sex was summarized in Supplementary Table 1.
Population characteristics	The mean age of cases was 37.45y ± 9.87. The mean preoperative Eckardt score was 7(range 5-9), and median disease duration was 2 years (range 0.33-6years). The mean age of controls 49.25y ± 10.44. Detailed clinical characteristics are summarized in Supplementary Table 1.
Recruitment	Participants admitted to Zhongshan Hospital with achalasia or benign leiomyomas that originated from LES were recruited in this study without selection bias. The inclusion and exclusion criteria were as follows.<br>Patients with achalasia were diagnosed by high-resolution manometry (HRM), combined with history, gastroenterological endoscope, and barium esophagogram. HRM was evaluated by the Chicago Classification Criteria.<br>Control LES tissue specimens were taken from age-matched patients undergoing submucosal tunneling endoscopic resection for benign leiomyomas that originated from LES to ensure no invasion of the normal tissue. After the leiomyomas removed, the control tissue specimens were taken from the surrounding normal tissue without tumor invasion.<br>Exclusion criteria of participants included: cardiovascular, metabolic, hematologic, infectious, inflammatory, neoplastic or autoimmune disease, and history of surgery, radiotherapy or immunotherapy.
Ethics oversight	This study was approved by the Research and Ethical Committee of Zhongshan Hospital, Fudan University and complied with all relevant ethical regulations. Written informed consent was provided by all participants.

Note that full information on the approval of the study protocol must also be provided in the manuscript.

Field-specific reporting

Please select the one below that is the best fit for your research. If you are not sure, read the appropriate sections before making your selection.

[x] Life sciences [ ] Behavioural & social sciences [ ] Ecological, evolutionary & environmental sciences

For a reference copy of the document with all sections, see nature.com/documents/nr-reporting-summary-flat.pdf

Life sciences study design

All studies must disclose on these points even when the disclosure is negative.

Sample size	Sample size was not predetermined at the time of recruitment. Available samples at the time of initiation of study were processed for sequencing.
Data exclusions	Two samples (case7.blood and case12.blood) were removed in TCR-seq analysis and one sample (case7.blood) was removed in BCR-seq analysis, because almost no cells in these samples were retained after quality control.
Replication	Due to limited cell number from patients sample, there was no replication of scRNA-seq experiment. Instead, we validated scRNA-seq results were validated by multicolor IHC and cyTOF.
Randomization	Randomization was not relevant to this study. Groups (case and control) were determined by clinical characteristics of donors.
Blinding	Blinding was not relevant, as investigators should group the donors based on clinical characteristics.

Reporting for specific materials, systems and methods

We require information from authors about some types of materials, experimental systems and methods used in many studies. Here, indicate whether each material, system or method listed is relevant to your study. If you are not sure if a list item applies to your research, read the appropriate section before selecting a response.

Materials & experimental systems

n/a	Involved in the study
☐	☒ Antibodies
☒	☐ Eukaryotic cell lines
☒	☐ Palaeontology and archaeology
☒	☐ Animals and other organisms
☒	☐ Clinical data
☒	☐ Dual use research of concern

Methods

n/a	Involved in the study
☒	☐ ChIP-seq
☒	☐ Flow cytometry
☒	☐ MRI-based neuroimaging

Antibodies

Antibodies used

Anti-PGP9.5 (clone EPR4118) (1:400) (Abcam, Cat# ab108986);
Anti-CD3 (clone /) (1:100) (Abcam, Cat# ab5690);
Anti-CD4 (clone BP6028) (1:100) (Biolynx, Cat# BX50023);
Anti-CD8 (clone 1G2B10) (1:4000) (Protrintech, Cat# 66868-1-AP);
Anti-CD69 (clone EPR21814) (1:500) (Abcam, Cat# ab233396);
Anti-CD68 (clone /) (1:1000) (Servicebio, Cat# GB113150);
Anti-C1QC (clone EPR2984Y) (1:100) (Abcam, Cat# ab75756);
Anti-TMEM119 (clone /) (1:500) (Abcam, Cat# ab185333);
Anti-TIM4 (clone EPR22304-3) (1:100) (Abcam, Cat# ab222093);
Anti-P2RY12 (clone EPR23511-72) (1:500) (Abcam, Cat# ab254347);
HRP Anti-Rabbit IgG antibody, (1:200) (Abcam, Cat# ab288151).

Validation

All antibodies used are commercially available, and have been previously validated by the manufacturer and/or in publications.
Validation of all antibodies are as follows.
https://www.abcam.com/products/primary-antibodies/pgp95-antibody-epr4118-neuronal-marker-ab108986.html
https://www.abcam.com/products/primary-antibodies/cd3-antibody-ab5690.html
http://www.biolynxtec.com/products/antibody/cd4.html
https://www.ptglab.com/products/CD8A-Antibody-66868-1-Ig.htm
https://www.abcam.com/products/primary-antibodies/cd69-antibody-epr21814-ab233396.html
https://servicebio.com/goodsdetail?id=21393
https://www.abcam.com/products/primary-antibodies/c1qc-antibody-epr2984y-ab75756.html
https://www.abcam.com/products/primary-antibodies/tmem119-antibody-c-terminal-ab185333.html
https://www.abcam.com/products/primary-antibodies/tim-4-antibody-epr22304-3-ab222093.html
https://www.abcam.cn/products/primary-antibodies/p2y12-antibody-epr23511-72-ab254347.html
